# OCT demonstrating neoatherosclerosis as part of the continuous process of coronary artery disease

**DOI:** 10.1007/s00059-015-4343-y

**Published:** 2015-08-11

**Authors:** B.-C. Zhang, A. Karanasos, E. Regar

**Affiliations:** Department of Cardiology, Thorax Center, Erasmus Medical Center, Room Ba-585, ‘s-Gravendijkwal 230, 3015 Rotterdam, The Netherlands; Department of Cardiology, The Affiliated Hospital of Xuzhou Medical College, 221002 Jiangsu, China

**Keywords:** Atherosclerosis, Optical coherence tomography, Percutaneous coronary intervention, Drug-eluting stents, Bare metal stents, Atherosklerose, Optische Kohärenztomographie, Perkutane Koronarintervention, Medikamentenbeschichtete Stents, Reine Metallstents

## Abstract

Although the advent of drug-eluting stents has reduced the rates of target vessel revascularization, there are observations of ongoing stent failure occurring very late after stent implantation and presenting as very late restenosis or as very late stent thrombosis. The de novo development of atherosclerosis within the neointimal region, called neoatherosclerosis, has been identified as one of the pathomechanisms of these observed late stent failures. The mechanisms of neoatherosclerosis development and its association with stent failure are currently the subject of intensive research. Optical coherence tomography (OCT) is an invasive imaging modality that allows us to visualize the micromorphology of coronary arteries with near-histological resolution, thus providing detailed assessment of the morphological characteristics of the neointima after stent implantation, including neoatherosclerosis. Several OCT studies have tried to provide in vivo insights in the mechanisms of neoatherosclerosis development and its association with late stent failure. This review summarizes the current insights into neoatherosclerosis obtained with OCT and discusses the association of neoatherosclerosis with late stent failure.

## Zusammenfassung

Zwar ist es durch das Aufkommen medikamentenbeschichteter Stents zu einer Senkung der Rate von Zielgefäßrevaskularisierungen gekommen, aber es wurden Fälle fortlaufenden Stentversagens beobachtet, die erst sehr spät nach Stentimplantation auftreten und sich mit großer zeitlicher Verzögerung als Restenosierung oder als sehr späte Stentthrombose manifestieren. Das De-novo-Auftreten einer Atherosklerose innerhalb der neointimalen Region, als Neoatherosklerose bezeichnet, hat sich als einer der Pathomechanismen dieser späten Fälle von Stentversagen herausgestellt. Die Mechanismen der Neoatheroskleroseentstehung und ihr Zusammenhang mit einem Stentversagen werden derzeit noch intensiv untersucht. Die optische Kohärenztomographie (OCT) ist ein invasives bildgebendes Verfahren, mit dem sich die Mikromorphologie der Koronararterien in nahezu histologischer Auflösung darstellen lässt und das somit eine detaillierte Beurteilung der morphologischen Merkmale der Neointima nach Stentimplantation, einschließlich Neoatherosklerose, ermöglicht. Im Rahmen verschiedener OCT-Studien wurde versucht, in vivo einen Einblick in die Mechanismen der Neoatheroskleroseentstehung und ihren Zusammenhang mit spätem Stentversagen zu erhalten. In der vorliegenden Übersichtsarbeit wurde der Schwerpunkt auf die zusammenfassende Darstellung aktueller Erkenntnisse aus der OCT in Bezug auf die Neoatherosklerose gelegt und der Zusammenhang der Neoatherosklerose mit spätem Stentversagen erörtert.

Drug-eluting stents (DES) have reduced the rates of target vessel revascularization and comprise the mainstay of treatment in percutaneous coronary intervention. However, despite the excellent short- and mid-term results with the current DES generation, ongoing stent failure with both bare metal stents (BMS) and DES is a frequent finding very late after stent implantation [1–3]. Very late stent failure clinically manifests as very late restenosis or stent thrombosis. Although the pathogenesis of (very) late stent failure appears to be multifactorial [4, 5], one of the major mechanisms that have been implicated is the de novo development of atherosclerosis within the neointimal region, called neoatherosclerosis [6, 7]. Observations of neoatherosclerosis have been documented both in ex vivo pathological observations and in vivo by intravascular imaging.

Among several imaging modalities that have been used to identify neoatherosclerosis [8–11], optical coherence tomography (OCT) can provide the most comprehensive assessment of the neointimal tissue. OCT allows for the visualization of the micromorphology of coronary arteries with near-histological resolution, differentiating between individual plaque components and providing important quantitative plaque information as the thickness of the fibrous plaque [12, 13]. Thus, by OCT it is possible to assess distinct morphological characteristics of neoatherosclerosis, such as macrophage infiltration, lipid accumulation, in-stent calcification, or neointimal rupture. Consequently, in vivo OCT studies have focused on studying in-stent neoatherosclerosis and its association with late stent failure. This review summarizes the current insights into neoatherosclerosis obtained with OCT and discusses the implications of neoatherosclerosis on long-term outcome after stent implantation.

## Definition and OCT imaging evidence of in-stent neoatherosclerosis

**Fig. 1:**
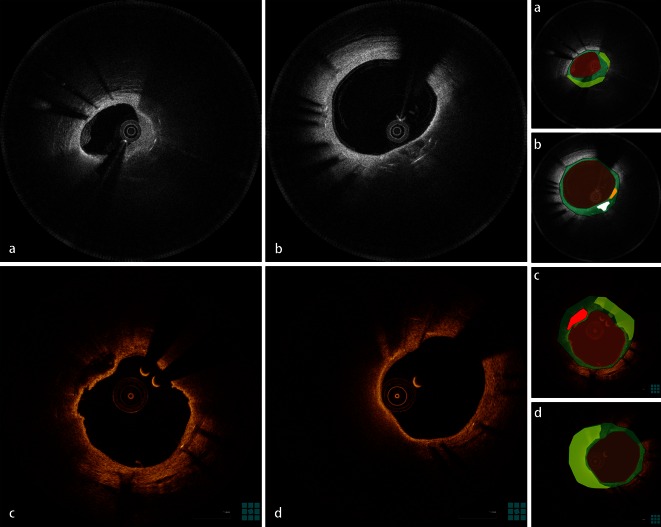
Morphological characteristics of in-stent neoatherosclerosis on optical coherence tomography images. **a** In-stent necrotic core (*yellow*) within the neointima (*green*). **b** In-stent calcification (*white*) and macrophage infiltration (orange). **c** Neointimal rupture. **d** In-stent necrotic core (*yellow*) with thin overlying fibrous cap. Panels on the right are color-coded cartoons of the corresponding OCT images, explaining the composition of the neointimal tissue.

Neoatherosclerosis refers to an atherosclerotic change in neointimal tissue, first described in pathologic specimens of BMS, and more recently in pathologic specimens of DES as well [6, 7]. Although observations of neoatherosclerosis had been sporadically documented from the early application of OCT [14, 15], the tissue properties of the observed neointimal tissue patterns were unknown. Pathological studies were the first to provide more comprehensive insights regarding the histological composition of this entity. These studies defined neoatherosclerosis as the presence of clusters of lipid-laden foamy macrophages with or without necrotic core formation and/or calcification within the neointimal tissue of stented segments [6]. The presence of morphological components of native atherosclerosis within the neointimal tissue implies that these features can be identified by OCT, which has a high accuracy for detection of these characteristics in native atherosclerotic plaques. This hypothesis has been corroborated by a pathologic study of ex vivo stented coronary arteries, imaged by OCT, which showed that the OCT appearance of these characteristics is similar to that in native atherosclerosis [16]. Components of neoatherosclerosis that can be visualized by OCT include macrophage infiltration, necrotic core, in-stent calcifications, and neoatherosclerotic plaque rupture [17]. An in-stent necrotic core visualized by OCT is defined as the presence of signal-poor, highly attenuating regions with poorly delineated borders within the neointima, while in-stent calcifications are defined as well-delineated, signal-poor regions with sharp borders [16]. In accordance with native atherosclerosis, macrophages on OCT appear as a thin signal-bright layer causing high attenuation, while neointimal plaque rupture appears as a discontinuation in the luminal surface with formation of a cavity burrowing into the neointima [16]. Representative OCT images of in-stent neoatherosclerosis are shown in Fig. 1.

**Fig. 2:**
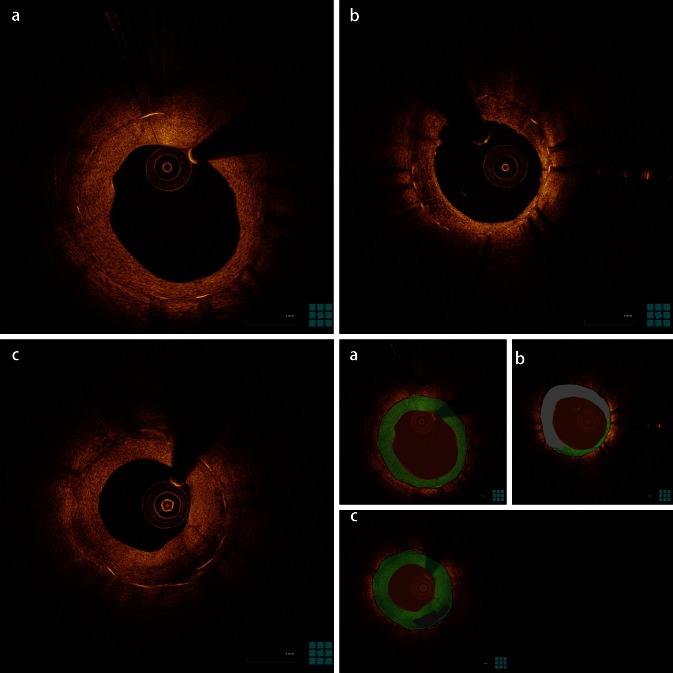
Patterns of neointimal coverage as visualized on optical coherence tomography images. **a** Homogeneous pattern (*green*); **b** heterogeneous pattern (*light gray*); **c** layered pattern (*dark gray*). Panels on the bottom right are color-coded cartoons of the corresponding OCT images, explaining the composition of the neointimal tissue.

At this point it is important to emphasize the difference of neoatherosclerosis with the previously reported heterogeneous and layered patterns of coverage [18]. These patterns are respectively defined as neointimal tissue with focally changing optical properties and varying backscattering patterns, and as neointimal tissue with concentric layers having different optical properties (an adluminal high scattering layer and an abluminal low scattering layer). Such patterns are often encountered in the context of in-stent restenosis, and although the pathological substrate is not well characterized, histological findings from atherectomy specimens, autopsy, and animal experiments include organized thrombus, fibrin, and myxomatous extracellular matrix with sporadic evidence of inflammation [16, 19–21]. The association of these patterns with neoatherosclerosis is yet unknown, however there is evidence that these patterns similar to the homogeneous pattern may subsequently be replaced by neoatherosclerotic tissue [22, 23]. Representative OCT images of different neointimal coverage patterns are shown in Fig. 2.

Although OCT can discern the distinct morphological characteristics of neoatherosclerosis, caution should be applied to image interpretation. This is because macrophage infiltration can sometimes appear as in-stent necrotic core, and vice versa [24]. Also, the distinction of a layered pattern of coverage with in-stent necrotic core can also be challenging sometimes, although the low attenuation observed in the case of the layered pattern can be used to discriminate between these two entities [16]. Finally, with OCT it is not possible to distinguish between true adluminal atherosclerosis from the progression of an abluminal underlying necrotic core into the neointima. Nevertheless, the latter mechanism appears to be less prevalent [25], while it is unclear what the clinical significance of such a discrimination would be.

## In vivo prevalence of neoatherosclerosis in BMS and DES with OCT

Several studies have tried to investigate the prevalence of neoatherosclerosis late after BMS or DES implantation using OCT (Table 1). The reported prevalence varies highly between the studies. As these studies differ significantly in stent type, in the interval between stent implantation and follow-up, and in the clinical presentation, this difference in prevalence appears to reflect differences in the composition of the studied population. The first reports of in-stent neoatherosclerosis report a prevalence of 67 % in patients with BMS implantation beyond 5 years [15]. Subsequent studies have shown lower prevalence ranging between 30 and 50 % in long-term follow-up of asymptomatic patients with BMS [26–28]; however, this incidence could be as high as 100 % in BMS restenosis more than 10 years since implantation [29]. With regard to neoatherosclerosis within DES, a prevalence of more than 50 % has been reported in a very late follow-up [27, 28], which can be even higher in stents with failure [14]. These rates tend to generally be in accordance with rates reported by pathological studies, which show a prevalence of 13–65 % depending on stent type and stent age [25].

**Table 1:** Reported prevalence of neoatherosclerosis assessed by OCT in late stent follow-up studies

Study	Indication (*n*)	Stent type (*n*)	Follow-up duration	Prevalence (%)
Takano et al. [15]	Follow-up (21)	BMS (21)	≥ 5 years	67
Kitabata et al. [27]	Follow-up (36)	1st and 2nd DES (19) BMS (17)	60 months 126 months	59 42
Kim et al. [22]	Follow-up (76)	1st and 2nd DES (76)	9 months 2 years	15 28
Kozuki et al. [23]	Follow-up (62)	1st DES (62)	3–12 months 36–80 months	3 23
Yonetsu et al. [30]	Follow-up (138)	1st and 2nd DES (82) BMS (56)	9 months 9–48 months ≥ 48 months 9 months 9–48 months ≥ 48 months	37 63 75 8 28 77
Kuramitsu et al. [34]	Follow-up (33)	2nd DES (12) 1st DES (11) BMS (10)	5 years	2 (frames) 10 (frames) 2 (frames)
Kitabata et al. [41]	Follow-up (46)	BMS (46)	≥ 4 years	47
Tian et al. [36]	Follow-up (109)	DES (109)	1 year	13.8
Hou et al. [26]	Follow-up (60)	BMS (60)	7 ± 1 years	33
Kang et al. [9]	Restenosis (50)	1st and 2nd DES (50)	32 (9–52) months	90
Kang et al. [29]	Restenosis (22)	BMS (22)	132 ± 31 months	100
Ino et al. [38]	Restenosis (48)	1st DES (48)	8 ± 1 months 34 ± 14 months	27 83
Ali et al. [8]	Restenosis (65)	1st and 2nd DES (51) BMS (14)	33 (16–60) months 36 (15–113) months	68 36
Habara et al. [45]	Restenosis (86)	1st DES (86)	1 year 1–3 years 3 years	2 14 35
Lee et al. [33]	Restenosis (212)	1st DES (111) 2nd DES (101)	55 months 12 months	46 11
Ko et al. [54]	Stent thrombosis (18)	1st and 2nd DES (18)	42 ± 21 months	22
Amabile et al. [50]	Stent thrombosis (20)	1st DES (4) and BMS(16)	8 (1–18) years	50
Amioka et al. [51]	Stent thrombosis (23)	1st DES (13) BMS (10)	1,750 ± 770 days 3,224 ± 1,380 days	46 50
Kang et al. [52]	Stent thrombosis (33)	1st DES (27) BMS (6)	Median: 62 months Median: 110 months	56 100
Alfonso et al. [67]	Stent thrombosis (15)	BMS(8) and DES (7)	Median: 347 days	27
Karanasos et al. [49]	Follow-up (22) Restenosis (13) Thrombosis (39)	BMS (13)/1st and 2nd DES (61)	≥ 18 months Median: 63 months	32 85 67

*BMS* bare metal stents, *1st DES* first-generation drug-eluting stents, *2nd DES* second-generation drug-eluting stents, *OCT* optical coherence tomography.

## Major factors associated with in-stent neoatherosclerosis

### Stent type and age

Pathological studies were the first to indicate a potential association of stent type and stent age with the prevalence of neoatherosclerosis [6]. DES appear to have a higher prevalence of neoatherosclerosis compared with BMS. Moreover, irrespective of stent type, the interval from implantation appears to be strongly associated with neoatherosclerosis development, an interval which is much lower than the interval required for development of native atherosclerotic plaques [25]. Nakazawa et al. [6] first demonstrated an increased prevalence of neoatherosclerosis in autopsy specimens of first-generation DES compared with BMS despite a shorter interval from implantation. Importantly, in both groups, the prevalence of neoatherosclerosis increased with the interval since implantation [6]. In vivo OCT studies have been in line with pathological studies showing an increased prevalence of neoatherosclerosis in DES at a follow-up of up to 4 years, although at a very long-term follow-up the prevalence is similarly high for both stent types [30]. Furthermore, serial imaging observations in first- and second-generation DES demonstrate that neointimas with a homogeneous or heterogeneous pattern of coverage might develop neoatherosclerosis over time with the prevalence of neoatherosclerosis increasing from 15 % at 9 months to 28 % at 24 months since implantation [22]. This was further corroborated by another serial imaging study in first-generation DES where the incidence of neoatherosclerosis increased from 3 % at mid-phase observations (3–12 months after stent implantation) to 23 % at late-phase observations [23].

Although DES in general appear to have a higher prevalence of neoatherosclerosis than BMS, it is not clear whether the prevalence of neoatherosclerosis differs between first- and second-generation DES. Second-generation DES have improved biocompatibility due to thinner struts and more biocompatible or bioabsorbable polymers, which translates to lower vascular toxicity and hypersensitivity reactions [31, 32]. However, a human autopsy study did not demonstrate any significant difference in the prevalence of neoatherosclerosis between a second-generation DES, cobalt-chromium everolimus-eluting stent (CoCr-EES), and first-generation paclitaxel-eluting (PES) or sirolimus-eluting stent (SES): CoCr-EES = 29 %; SES = 35 %; PES = 19 % [32]. Conversely, an in vivo OCT study in 212 DES-treated patients with > 50 % stenosis (101 patients had a first-generation and 111 patients had a second-generation DES) demonstrated a significantly lower prevalence of neoatherosclerosis in the second-generation DES group (10.8 vs. 45.5 %, *p* < 0.001); however, this difference was not significant after adjusting for time since implantation [33]. Similar findings were observed with OCT in a comparison of the coronary arterial response in biodegradable polymer biolimus-eluting stents (BES) versus durable polymer SES and BMS at the 5-year follow-up. The number of cross sections with neoatherosclerosis within BES was similar to BMS (BES = 2.26 % vs. BMS = 2.23 %, *p* = 0.98) and tended to be lower than SES (BES = 2.26 % vs. SES = 9.90 %, *p* = 0.07) [34].

The reasons why the temporal course of atherosclerosis within stents is accelerated are not well understood; however, dysfunction of endothelial cells, which are barriers that prevent lipid infiltration and migration of inflammatory cells, has been considered to be the main mechanism. This mechanism can also explain the increased prevalence of neoatherosclerosis observed in DES, where local drug delivery inhibits neointimal hyperplasia, causing delayed coverage and dysfunction of endothelial cells [35].

### Clinical factors

In addition to the stent type and age, several patient characteristics have been associated with neoatherosclerosis. Two registries of late stent follow-up have both identified chronic kidney disease as a factor independently associated with neoatherosclerosis [28, 33]. Other identified factors included low-density lipoprotein cholesterol > 70 mg/dl [33], current smoking, and lack of treatment with angiotensin-converting enzyme inhibitors or angiotensin II receptor blockers [28]. In another study, the presence of diabetes mellitus was associated with a higher incidence of neoatherosclerosis at a late DES follow-up (18.3 vs. 5.5 %, *p* = 0.03) [36]. Further subgroup analysis showed that elevated glycated hemoglobin (HbA_1c_) levels (> 7 %) in patients with diabetes mellitus were associated with a higher incidence of neoatherosclerosis (28 vs. 7 %, *p* = 0.048).

### Plaque and stent characteristics

Plaque characteristics have also been implicated in the neoatherosclerotic process. Microvessels play a key role in advanced atherosclerosis, which is closely associated with plaque hemorrhage and plaque rupture [37], while previous investigations have demonstrated a higher incidence of microvessels in late in-stent restenotic tissue, suggesting that neovessels might be a trigger for in-stent neoatherosclerosis [38, 39]. Moreover, a spatial correspondence of neoatherosclerosis and microvessels was identified at a follow-up OCT examination of BMS and DES [40], supporting the hypothesis of microvessel involvement in neoatherosclerosis development. In the same study, neoatherosclerosis was more frequently identified in the proximal and distal stent sections, also associated with the morphology of the adjacent vessel segment. Specifically, the presence of lipid plaque in the adjacent stent edges was associated with the presence of neoatherosclerosis at the stent edges, implying that progression of native disease might also contribute to neoatherosclerosis. Apart from plaque characteristics, stent characteristics such as strut thickness seem to play a role even among the same stent type. In a study assessing the prevalence of neoatherosclerosis in BMS at a long-term follow-up (≥ 4 years after implantation), thick-strut stents (≥ 100 μm) had a higher prevalence of neoatherosclerosis compared with thin-strut stents (70 vs. 32 %, *p* = 0.02) [41].

### Neoatherosclerosis and in-stent restenosis

**Fig. 3:**
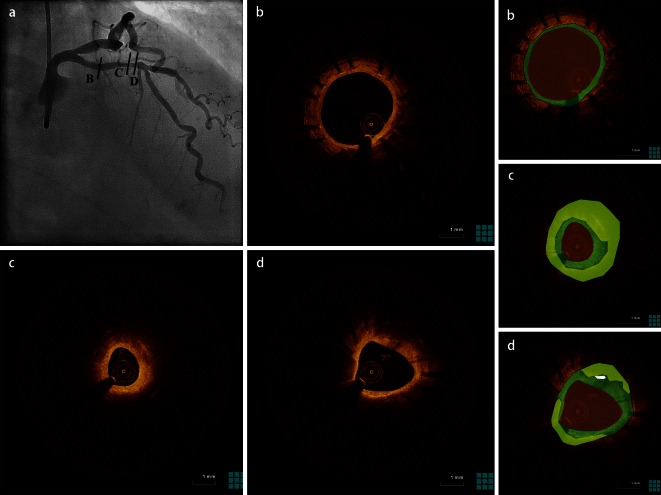
Late restenosis due to neoatherosclerosis. **a** Angiogram of patient presenting with stable angina 15 years after sirolimus-eluting stent implantation reveals a possible restenotic lesion in the site of the previously implanted stent. Optical coherence tomography study reveals a homogeneous pattern of coverage (*green*) proximal to the stent (**b**), but with neoatherosclerosis development (*yellow*) at the distal segment (**c** and **d**) causing lumen compromise. Panels on the right are color-coded cartoons of the corresponding OCT images, explaining the composition of the neointimal tissue.

Although early in-stent restenosis mainly results from aggressive neointimal proliferation [42], recent data also suggest that neoatherosclerosis may play an important pathophysiological role, especially in late restenosis. Pathological studies have demonstrated that neoatherosclerosis represents a common substrate in patients with late stent failure [43], while in vivo OCT studies have further elucidated the role of neoatherosclerosis in the development of late stent failure (Fig. 3). The presence of neoatherosclerosis has been associated with a higher degree of neointimal hyperplasia, independent from stent type and time since implantation [44]. Although during the early phases after DES implantation, homogeneous or heterogeneous patterns of coverage are the predominant pathology in in-stent restenosis, in later follow-up intervals restenosis is often associated with the presence of neoatherosclerosis [38, 45]. A study focusing on 50 patients with DES restenosis at a median follow-up of 32 months since implantation showed a prevalence of neoatherosclerosis of 52 % [9]. Also, in lesions with very late BMS restenosis beyond 10 years since implantation, neoatherosclerosis was detected in 100 % of the stents with a high frequency of neointimal rupture and neointimal thrombi [29]. However, a comparison of restenotic lesions of DES and BMS showed that neoatherosclerosis occurs earlier in DES compared with BMS and develops more diffusely along stented vessels with thinner cap and greater total lipid core [8]. Whether the contribution of neoatherosclerosis in restenosis of second-generation DES is similar to that of first-generation DES is not well established; however, observations of restenosis due to neoatherosclerosis have also been reported for second-generation DES [33].

### Neoatherosclerosis and stent thrombosis

**Fig. 4:**
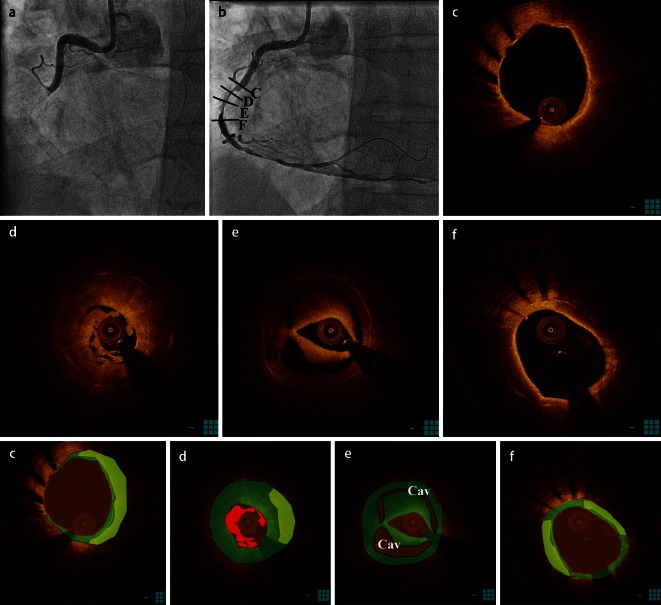
Very late thrombosis due to neoatherosclerosis. **a** Angiogram of patient presenting with ST-elevation myocardial infarction 7 years after everolimus-eluting stent implantation in the right coronary artery showing total occlusion. After thrombus aspiration an in-stent lesion is obvious (**b**), while optical coherence tomography study shows neoatherosclerosis development (*yellow*) with neointimal rupture leading to cavity formation (*Cav*) and thrombus (*red*) (**c**–**f**). Homogeneous neointima is indicated in *green.* Panels on the bottom are color-coded cartoons of the corresponding OCT images, explaining the composition of the neointimal tissue.

Neoatherosclerosis also appears to contribute to the development of (very) late stent thrombosis (Fig. 4). Initial pathological studies demonstrated a role for the underlying plaque in BMS thrombosis, while subsequent studies in DES incriminated an impaired healing response with high rates of uncovered struts and vascular toxicity-associated malapposition as the main pathogenetic factor [46, 47]. Nevertheless, more recent pathological and intracoronary imaging data support the hypothesis that neoatherosclerosis is an active causal mechanism in a high percentage of cases with late stent thrombosis [32, 48, 49]. Autopsy studies have shown that thrombosis might develop on the grounds of neointimal plaque rupture [32], while thrombus aspirate samples from patients with very late BMS thrombosis demonstrate the presence of atherosclerotic plaque components such as foamy macrophages and cholesterol crystals within the aspirates [48]. Simultaneously, a number of studies have examined the OCT findings in patients with late and very late stent thrombosis, in an attempt to identify the pathomechanisms [50–54]. Despite minor discrepancies in the exact prevalence of neoatherosclerosis and the relative contribution of neointimal plaque rupture in each study, these studies collectively demonstrate a very high prevalence of plaque rupture as a pathomechanism for very late BMS thrombosis [55], and an almost equal contribution for neointimal plaque rupture and impaired healing in (very) late DES thrombosis [50, 52, 53]. Compared to an impaired healing response, neointimal rupture is a mechanism occurring mainly at longer intervals since stent implantation [50, 56], in neointimal regions with high necrotic core content and thin fibrous cap, similarly to what is observed in native atherosclerosis, but also with a very high incidence of macrophage infiltration [49].

**Fig. 5:**
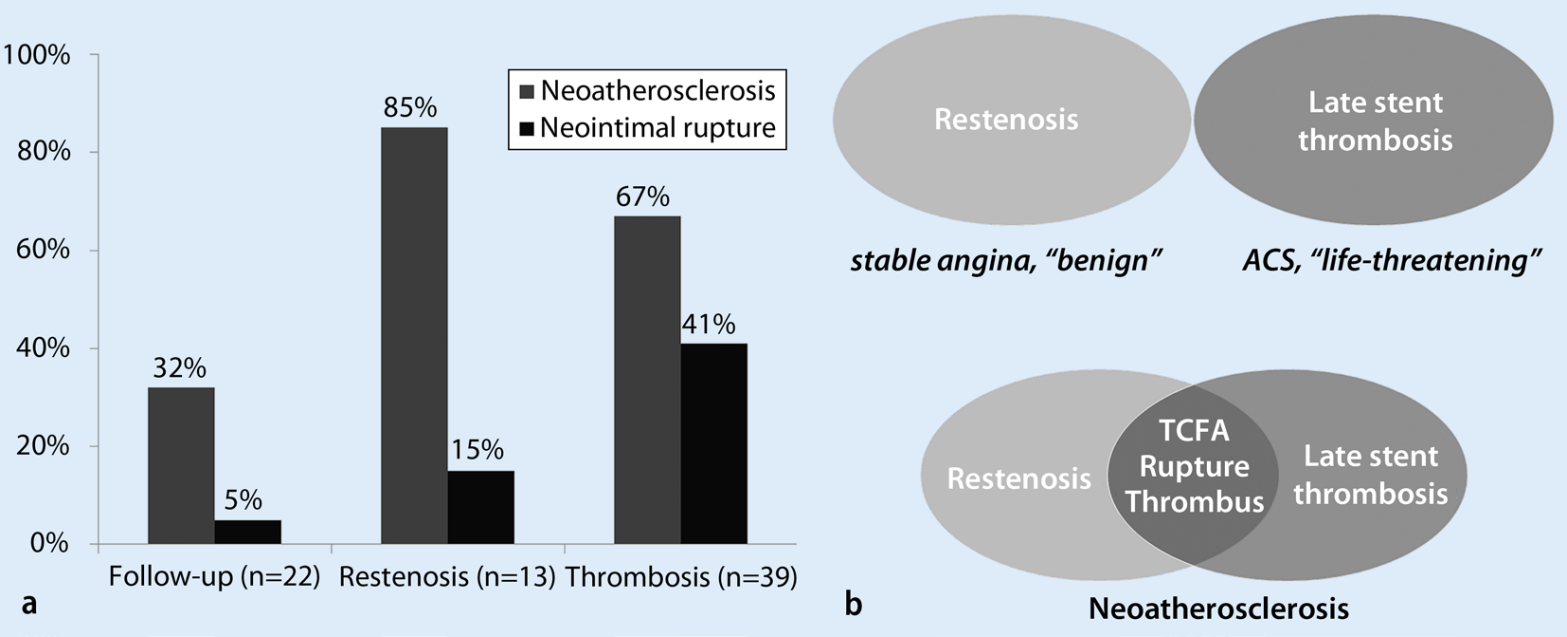
**a** Prevalence of neoatherosclerosis and neointimal rupture according to clinical presentation [49]. **b** Change in the understanding of stent failure by intravascular imaging insights. Although stent restenosis had traditionally been considered a benign entity associated with stable symptoms and stent thrombosis had been considered a life-threatening entity associated with acute presentation, intravascular imaging has disclosed the participation of neoatherosclerosis in both entities, with several imaging findings such as in-stent necrotic core, neointimal rupture, and thrombus being common between them

Evidence also suggests that the clinical presentation of late stent failure is not exclusively affected by the presence of neoatherosclerosis but also by the morphological characteristics. In patients with in-stent restenosis, a higher incidence of OCT-defined thin-cap fibroatheroma, intimal rupture, and thrombi was identified in patients presenting with unstable angina compared with patients with stable symptoms [9]. Our group investigated the differences in the prevalence of neoatherosclerosis with regard to the clinical presentation [49]. Both stent restenosis and stent thrombosis had a higher prevalence of neoatherosclerosis compared with the asymptomatic group (stent thrombosis: 67 % vs. restenosis: 85 % vs. asymptomatic: 32 %; *p* < 0.001). However, the prevalence of neointimal rupture was higher in the stent thrombosis group (stent thrombosis: 41 % vs. restenosis: 15 % vs. asymptomatic: 5 %; *p* < 0.001) (Fig. 5a). This finding underscores the contribution of neoatherosclerosis development in the pathogenesis of late stent failure, while it indicates that neointimal rupture is mainly associated with acute presentation, although not infrequently encountered in stable or asymptomatic in-stent lesions.

Overall, intravascular imaging has changed the understanding of the pathogenesis of late restenosis and thrombosis by disclosing that neoatherosclerosis is a common pathomechanism in both entities (Fig. 5b). Stent restenosis had traditionally been considered a rather benign entity associated with stable symptoms, while stent thrombosis was seen as a life-threatening entity associated with acute presentation, with these two entities considered to be associated with different pathogenetic substrates. However, the presence—to a different extent—of common imaging findings such as in-stent necrotic core, neointimal rupture, and thrombus in both entities implies that these two presentations can occur as different manifestations of the same unfavorable healing process.

### Neoatherosclerosis and bioresorbable scaffolds

**Fig. 6:**
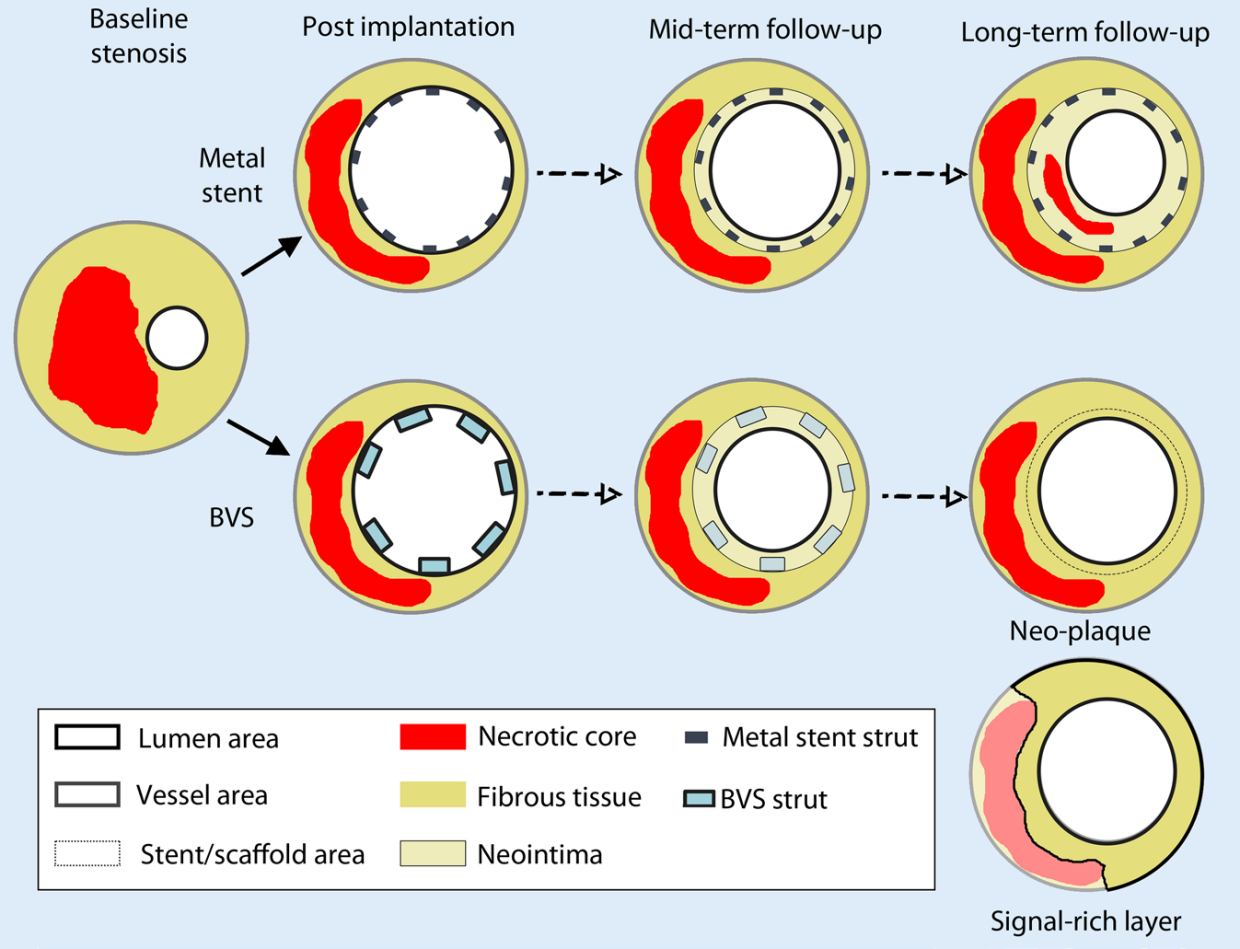
Paradigm of healing response in metal stents and bioresorbable scaffolds. After metal stent implantation, struts are preserved and the neointimal area is clearly delineated between the stent and lumen contour even at long-term follow-up, with possible development of neoatherosclerosis within the neointima. Conversely, in long-term follow-up of bioresorbable scaffolds, neointimal boundaries are unclear after bioresorption (*dotted line*), and the intima resembles a native plaque, defined as neoplaque. The signal-rich layer is the layer that separates the underlying plaque components from the lumen. *BVS* bioresorbable vascular scaffold. (Adapted from Karanasos et al. [57])

Bioresorbable vascular scaffolds (BVS) are a new technology for percutaneous revascularization that are resorbed after an interval of 2–4 years since implantation. Conceptually, after this interval, the permanent vessel caging with the associated endothelial dysfunction disappears and this can lead to a reduction of stent-induced complications such as hypersensitivity reactions or neoatherosclerosis. First-in-man studies of BRS have demonstrated a favorable healing response at a very long-term follow-up with complete strut reabsorption, late luminal enlargement, and a potentially favorable plaque modification, while no cases of necrotic core accumulation of adluminal origin were observed [57]. As after bioresorption, the area corresponding to struts and neointima is consolidated with the underlying plaque, it resembles a native atherosclerotic plaque that is now well separated by the lumen by a signal-rich layer, a process modulated by hemodynamic factors [58] (Fig. 6). Nevertheless, as these first-in-man studies have focused on simple lesions, data on more complex lesions including thrombotic lesions are scarce [59]. Studies on more complex populations are ongoing, without, however, any worrying signs regarding neoatherosclerosis development in human studies of BVS thus far. Moreover, studies reporting on (very) late BVS thrombosis have not shown any evidence of scaffold thrombosis due to neointimal plaque rupture [60]. Therefore, the further development and implementation in the clinical practice of bioresorbable technologies might allow these complications to be overcome, although more long-term data are needed to confirm a potential benefit of this technology.

## Newer OCT techniques for assessment of neoatherosclerosis

As previously mentioned, OCT is associated with some limitations in assessment of neoatherosclerosis. Nevertheless, new developments in OCT could improve in-stent tissue characterization and provide an enhanced and more objective neointimal tissue characterization. One of these developments is quantitative attenuation imaging. Tissue properties such as attenuation have been shown in ex vivo studies to be associated with the presence of macrophages and/or necrotic core [61, 62], while in vivo studies have demonstrated the potential of this technique for the detection of lipid plaques and fibroatheromas [63]. Therefore, this technique could be used for the assessment of neoatherosclerosis, since the optical properties of tissue components such as macrophages and a necrotic core are similar between native atherosclerosis and neoatherosclerosis. OCT assessment of neoatherosclerosis can also be improved through software for automated fibrous cap thickness measurement that can reduce human variability in the assessment of in-stent fibroatheromas [64]. Furthermore, the application of technologies such as polarization-sensitive OCT in humans can provide further structural information such as collagen and smooth muscle content of neointimal tissue [65], while high-speed OCT catheters will be able to acquire images from an entire coronary artery in less than 1 s [66].

### Conclusion and perspectives

Recent evidence has highlighted the significance of in-stent neoatherosclerosis as a disease entity that comprises an important substrate for late adverse cardiac events after stent implantation and is one of the major pitfalls of the current generation of metallic stents. Pathological studies have demonstrated that infiltration of lipid-laden foamy macrophages with or without necrotic core and/or calcification formation constitutes the pathological substrate. A number of in vivo studies have used OCT—an imaging modality that can accurately identify morphological characteristics such as in-stent necrotic core, in-stent calcification, macrophage infiltration, and neointimal plaque rupture—in order to assess the in vivo characteristics and implications of this disease entity. These have demonstrated that neoatherosclerosis appears earlier and more frequently in drug-eluting stents compared with bare metal stents, is associated with several clinical and morphological factors, while its prevalence increases over time. Importantly, an association of neoatherosclerosis with late stent failure has been demonstrated, manifesting either as late restenosis, or in cases with neointimal plaque rupture as late stent thrombosis. Bioresorbable technologies hold promise for the reduction of this entity; however, their long-term healing response needs to be better documented. Further studies are needed so as to demonstrate what the prognostic implications of neoatherosclerosis detection are, and whether the natural history of this disease entity can be modified. Meanwhile, new devices for percutaneous revascularization need to be developed that can alleviate this entity, in order to be able to reduce the incidence of very late events after percutaneous coronary intervention.
